# Violent Deaths of Iraqi Civilians, 2003–2008: Analysis by Perpetrator, Weapon, Time, and Location

**DOI:** 10.1371/journal.pmed.1000415

**Published:** 2011-02-15

**Authors:** Madelyn Hsiao-Rei Hicks, Hamit Dardagan, Gabriela Guerrero Serdán, Peter M. Bagnall, John A. Sloboda, Michael Spagat

**Affiliations:** 1 Health Service and Population Research Department, Institute of Psychiatry, King's College, London, United Kingdom; 2 Iraq Body Count, London, United Kingdom; 3 Department of Economics, Royal Holloway, University of London, Egham, United Kingdom; 4 The MacMillan Center for International and Area Studies, Yale University, New Haven, Connecticut, United States of America; The University of Queensland, Australia

## Abstract

**Background:**

Armed violence is a major public health and humanitarian problem in Iraq. In this descriptive statistical analysis we aimed to describe for the first time Iraqi civilian deaths caused by perpetrators of armed violence during the first 5 years of the Iraq war: over time; by weapon used; by region (governorate); and by victim demographics.

**Methods and Findings:**

We analyzed the Iraq Body Count database of 92,614 Iraqi civilian direct deaths from armed violence occurring from March 20, 2003 through March 19, 2008, of which Unknown perpetrators caused 74% of deaths (*n* = 68,396), Coalition forces 12% (*n* = 11,516), and Anti-Coalition forces 11% (*n* = 9,954). We analyzed the subset of 60,481 civilian deaths from 14,196 short-duration events of lethal violence to link individual civilian deaths to events involving perpetrators and their methods. One-third of civilian violent death was from extrajudicial executions by Unknown perpetrators; quadratic regression shows these deaths progressively and disproportionately increased as deaths from other forms of violence increased across Iraq's governorates. The highest average number of civilians killed per event in which a civilian died were in Unknown perpetrator suicide bombings targeting civilians (19 per lethal event) and Coalition aerial bombings (17 per lethal event). In temporal analysis, numbers of civilian deaths from Coalition air attacks, and woman and child deaths from Coalition forces, peaked during the invasion. We applied a Woman and Child “Dirty War Index” (DWI), measuring the proportion of women and children among civilian deaths of known demographic status, to the 22,066 civilian victims identified as men, women, or children to indicate relatively indiscriminate perpetrator effects. DWI findings suggest the most indiscriminate effects on women and children were from Unknown perpetrators using mortar fire (DWI  = 79) and nonsuicide vehicle bombs (DWI  = 54) and from Coalition air attacks (DWI  = 69). Coalition forces had higher Woman and Child DWIs than Anti-Coalition forces, with no evidence of decrease over 2003–2008, for all weapons combined and for small arms gunfire, specifically.

**Conclusions:**

Most Iraqi civilian violent deaths during 2003–2008 of the Iraq war were inflicted by Unknown perpetrators, primarily through extrajudicial executions that disproportionately increased in regions with greater numbers of violent deaths. Unknown perpetrators using suicide bombs, vehicle bombs, and mortars had highly lethal and indiscriminate effects on the Iraqi civilians they targeted. Deaths caused by Coalition forces of Iraqi civilians, women, and children peaked during the invasion period, with relatively indiscriminate effects from aerial weapons.

*Please see later in the article for the Editors' Summary*

## Abstract 2

Madelyn Hsiao-Rei Hicks and colleagues provide a detailed analysis of Iraqi civilian violent deaths during 2003-2008 of the Iraq war and show that of 92,614 deaths, unknown perpetrators caused 74% of deaths, Coalition forces 12%, and Anti-Coalition forces 11%.

## Editors' Summary

**Background:**

Civilian deaths through armed violence is a public health and humanitarian problem in all wars, despite internationally agreed humanitarian standards regarding the treatment of civilians during wars—so-called laws of war such as the Geneva Conventions. Since the Iraq war began on March 20, 2003, when a multilateral force led by US and UK troops invaded Iraq, more than 100,000 Iraqi civilians (women, children, noncombatants, and police carrying out nonparamilitary duties) have died because of armed violence, according to the Iraq Body Count (IBC), a nongovernmental project that collates media reports of deaths of individual Iraqi civilians and cross-checks these reports with data from hospitals, morgues, nongovernmental organizations, and official figures. Indeed, according to a recent assessment of the global burden of armed violence, in 2006, people living in Iraq had the highest risk of dying violently in conflict. In that year, there were 91 violent deaths per 100,000 people in the country.

**Why Was This Study Done?:**

Detailed analysis of civilian deaths during wars is important because it can improve the understanding of the impact of these deaths on general public health and on vulnerable subgroups in the population. In addition, data collected on the nature and effects of violence can guide the development of preventative policies. For example, an analysis that reveals that air attacks by invading troops cause a high proportion of civilian deaths might encourage policy changes that prohibit air attacks on populated areas. Finally, by linking violent deaths to perpetrators, analyses of civilian deaths can provide an indicator of combatants' compliance with the laws of war, which require the protection of civilians from targeted or indiscriminate harm. Here, IBC researchers provide a descriptive statistical analysis of Iraqi civilian deaths directly caused by perpetrators of armed violence during the first 5 years of the Iraq war.

**What Did the Researchers Do and Find?:**

According to data from the Iraq Body Count, more than 92,000 Iraqi civilians died because of armed violence during this period. Coalition forces (identified by uniforms) caused 12% of these deaths, anti-coalition forces (un-uniformed combatants identified by attacks on coalition targets) caused 11% of the deaths; and unknown perpetrators, who targeted civilians and were indistinguishable from their victims (for example, a suicide bomber in a market), were responsible for three-quarters of civilian deaths. To link individual deaths with perpetrators and their methods, the researchers analyzed the 60,481 civilian deaths caused by short-duration events of lethal violence (events that lasted less than 24 hours and that occurred in a specific location; for example, overnight air strikes). Extrajudicial executions by unknown perpetrators were responsible for one-third of these deaths and disproportionately increased as deaths from other forms of violence increased across Iraq. Unknown perpetrator suicide bombings that targeted civilians and coalition aerial bombings killed most civilians per lethal event (19 and 17 deaths per lethal event on average, respectively). Finally, the researchers calculated the proportion of women and children among civilian deaths. Because men are the main targets of armed violence, this proportion—the “Dirty War Index” (DWI)—indicates the scale of indiscriminate killing in a conflict. The most indiscriminate effects on women and children in Iraq were from unknown perpetrators firing mortars (DWI  = 79) and nonsuicide vehicle bombs (DWI  = 54), and from coalition air attacks (DWI  = 69). Coalition forces had a higher DWI than anti-coalition forces for all weapons combined, with no decrease over the study period.

**What Do These Findings Mean?:**

These findings show that during the first 5 years of the Iraq war, civilian deaths varied over time and location and in terms of victim characteristics and targeting of civilians. Although limited to direct deaths and possibly subject to some media bias, these findings show that most civilian deaths were inflicted by unknown perpetrators, and that unknown perpetrators had particularly lethal and indiscriminate effects on Iraqi civilians. However, they also show that Coalition forces had indiscriminate lethal effects on civilian populations. In part, this may be because Coalition forces had a high risk of killing civilians accidentally because they could not easily recognize anti-coalition combatants fighting without uniforms among civilians. Nevertheless, the relatively indiscriminate effects of Coalition aerial weapons highlight the need to change policies relating to the use of air power in future armed conflicts.

**Additional Information:**

Please access these Web sites via the online version of this summary at http://dx.doi.org/10.1371/journal.pmed.1000415.

- This study is further discussed in a *PLoS Medicine* Perspective by Robert Muggah
- The International Committee of the Red Cross provides information about war and International humanitarian law (in several languages)
- The Geneva Declaration on Armed Violence and Development Web site provides information on the global burden of armed violence
- More details on the Iraq Body Count are available
- The Human Security Report Project tracks global and regional trends in organized violence, their causes, and consequences
- Every Casualty supports and is a resource for the documentation of individual casualties of armed conflict

## Introduction

Armed violence in war is an ongoing, significant public health and humanitarian problem internationally [1]–[9]. A global assessment of the burden of armed violence in 2004–2007 found that persons living in Iraq had the highest risk of dying violently in conflict, peaking at 91 violent deaths per 100,000 population in 2006 [7]. In 2009, we described patterns of civilian death caused by different weapons used in the Iraq war, and highlighted weapon-effects on children and female civilians [10]. Here, we analysed the perpetrators of armed violence in Iraq. We have also expanded our analysis to describe civilian deaths by perpetrators over time, by geographic area, to compare effects of weapons as used by different perpetrators, and to compare the demographic composition of their civilian victims.

Analysis of carefully documented civilian deaths caused by perpetrators and their weapons improves understanding of their impact on general public health as well as on vulnerable demographic subgroups, creates a burden of responsibility, and provides data on the nature and effects of violence to inform the development of preventive policies [1]–[12]. The Iraq war has involved both conventional state-to-state warfare and asymmetric, irregular warfare, with continuous international media coverage that has resulted in detailed reports on thousands of events causing civilian death. Iraq Body Count (IBC), a nongovernmental organization, has systematically collated a wide range of data from such reports as a means to monitor and document Iraqi civilian casualties from armed violence since the war's beginning on March 20, 2003 [13],[14]. The resulting database interlinks specific violent events with their perpetrators, the weapons used, the individual civilians killed, and the victim's demographic characteristics. By making links to perpetrators, civilian death can be examined not only as an important public health outcome, but also as an indicator of combatants' compliance with laws of war that require the protection of civilians from targeted or indiscriminate harm [7],[15]. Laws of war are international humanitarian laws and customary standards regarding the treatment of civilians and combatants (e.g., the Geneva Conventions) [15],[16].

Our specific aims in this study were: (1) to describe Iraqi civilian deaths attributable to perpetrators over time, specifically over the first 5 y of the Iraq war; (2) to identify which forms of armed violence used by various perpetrators caused the greatest numbers of civilian deaths or killed the most civilians in an average event in which a civilian died; (3) to describe the distribution of civilian violent deaths across Iraq's governorates and, because in 2009 we identified extrajudicial executions as the most prevalent form of violent death [10], to assess whether numbers of execution deaths in governorates had a relationship to numbers of nonexecution deaths and if so, what kind of relationship; (4) to describe the demographic composition of perpetrators' civilian victims over time and by weapon in terms of deaths of men, women, and children. We emphasize deaths of women and children because they are identified as vulnerable populations in public health and under laws of war [15],[16]. Moreover, because women and children are less often targeted than men in Iraq's conflict [10], as in most conflicts [17],[18], high proportions of women and children among victims can signal possible indiscriminate behavior or weapons [5],[10]. We measure proportional rates at which perpetrators in Iraq generated a particularly unacceptable public health and humanitarian outcome—killing women and children—by using a Woman and Child “Dirty War Index” (DWI). The DWI is a data-driven public health tool based on laws of war that can systematically identify rates of a particularly undesirable or prohibited, i.e., “dirty,” war outcome inflicted on populations during armed conflict [15]. Our final aim was to compare military opponents using a common weapon-type in war—small arms—to see if they differed in causing deaths of Iraqi civilians, women, and children by their gunfire.

## Methods

### The Database

The IBC database was prospectively developed by the authors HD and JAS when an invasion of Iraq appeared imminent in 2003, with the aim of systematically recording and monitoring deaths of individual Iraqi civilians from armed violence [13],[14]. Data sources are mainly professional media reports, including international and Iraqi press in translation. IBC uses key-word searches to scan Internet-published, English-language press and media reports of armed violence in Iraq directly resulting in civilian death. This process uses search engines and subscription-based press and media collation services (e.g., LexisNexis). Reports are scanned from over 200 separate press and media outlets meeting IBC's criteria: (1) public Web-access; (2) site updated daily; (3) all stories separately archived on the site, with a unique URL; and (4) English as a primary or translated language. Sources include dozens of Arabic-language news media that release violent incident reports in English (e.g., Voices of Iraq and Al Jazeera English), and report translation services such as the BBC Monitoring Unit. The three most frequently used sources are Reuters, Associated Press, and Agence France Presse. These and other international media in Iraq increasingly employ Iraqis trained in-house as correspondents. Media-sourced data are cross-checked with, and supplemented by, data from hospitals, morgues, nongovernmental organizations, and official figures [13].

For deaths to be added to the IBC database, at least one civilian must be reported killed by the event, with the number of deaths indicated in the source, and time and location adequately described for IBC to avoid double-counting. IBC has found that no single media source covers much more than half of all lethal violent incidents reported, thereby requiring a systematic process such as IBC's to knit together media and other sources in order to collate all reported violent deaths of civilians and to ensure that deaths are not double-counted. Whenever possible on the basis of reported data, IBC records a total of 18 variables for each lethal event, including: time of event, location of event, presence of an identifiable target, perpetrators, weapons, media sources, witnesses, injuries, and name, sex, age, and occupation of each victim. When accounts from independent sources differ, variables are entered from reports with the most detail or the best-placed informants (e.g., medical personnel attending to victims). Most frequent informants for reported armed violence deaths are morgue and hospital medics, police and other Iraqi official sources, eyewitnesses, and relatives. When equally credible reports differ on the number of deaths, minimum and maximum deaths are recorded for the event. Entries are independently reviewed and systematically error-checked by three IBC members before data are published on IBC's open Web site [13].

We analyzed the IBC database records updated as of July 12, 2010 for Iraqi civilian violent deaths that occurred during the first 5 y of the Iraq war, which began with the US-led Coalition invasion on March 20, 2003: March 20, 2003 through March 19, 2008. We first analyzed the total database, which included deaths from aggregate reports (hospital and morgue reports) and deaths from violent events of any duration, to describe yearly and total reported Iraqi civilian violent deaths by perpetrators. From this total database, we extracted a dataset of civilian deaths attributable to specific short-duration events. Short-duration events span no more than two calendar dates, occur in a specific location, and cause one or more reported direct civilian deaths (e.g., an overnight air strike, a brief gunfire ground-engagement, an isolated shooting). Analyzing short-duration events allows tighter, more reliable linkages between a perpetrator's use of a particular weapon-type in a specific time and place, and their effects on numbers killed and victim demographics. Use of limited-duration events also allows comparison of perpetrators' effects within a uniform time span (in this case, two days).

### Definitions

Iraqi civilian deaths as defined in the IBC database and this study include all children, most women, all noncombatants, and police carrying out regular, but not paramilitary, duties, as police constitute part of civil society [19]. A child is under age 18, based on the Convention on the Rights of the Child [20] and Iraqi law that 18 is the voting age and age of consent [14]. Women and men are adults aged 18 y or older, of known sex. Age was determined on the basis of reported age in years, or reported age category as “child” or “adult” or adult occupation. Execution is the extrajudicial killing of any abducted or captured individual by any method. Executions include combatants subjected to extrajudicial execution postcapture, as after capture they become noncombatants protected under international humanitarian law [15],[16].

### Perpetrators

Iraq's conflict environment is one in which perpetrators are not equally identifiable when they harm Iraqi civilians. Coalition forces are identifiable by uniforms, and in some cases (e.g., where aircraft are involved), by weapons. In contrast, sectarian and Anti-Coalition insurgent forces routinely do not wear uniforms or identifying marks during military actions [21]–[23]. Moreover, claims of responsibility for attacks, if made at all, are unreliable, and responsibility may be distributed across multiple groups due to the practice of subcontracting stages of weapon production and deployment [19],[21],[24]. IBC accurately reflects the nature of Iraq's armed conflict and the extent to which perpetrators of violence can, and cannot, be identified, through its three main perpetrator categories: Coalition forces, Anti-Coalition forces, and Unknown perpetrators. Deaths are attributed to Coalition forces (which chiefly consist of US forces) when data from reports identify Coalition perpetrators. Anti-Coalition forces, although visually indistinguishable from civilians, are identified as Anti-Coalition by their attack on a Coalition target (which includes Coalition-associated targets, such as Iraqi police checkpoints, Iraqi security forces, and government targets). Unknown perpetrators are those who target civilians (i.e., no identifiable military target is present), while appearing indistinguishable from civilians: for example, a suicide bomber disguised as a civilian in a market. Unknown (i.e., unidentified) perpetrators in Iraq include sectarian combatants and Anti-Coalition combatants who maintain a civilian appearance while targeting civilians, and criminals [19],[21]–[23].

### Statistical Analysis

Intercooled Stata 10.0 was used to calculate means, proportions, and regressions. Maximum deaths are used for events with reported minimum and maximum numbers because IBC finds subsequent evidence usually confirms initial maximum reports. Proportions were compared using chi-square testing, and means using one-way ANOVA, to obtain two-tailed *p*-values.

## Results

### Total Reported Civilian Violent Deaths over Time

Table 1 shows reported Iraqi civilian deaths from armed violence for the period of March 20, 2003 through March 19, 2008, by responsible perpetrator and by postinvasion year. Among the total 92,614 Iraqi civilian deaths from armed violence documented for 2003–2008, Unknown perpetrators caused 74% of all violent deaths of civilians (*n* = 68,396); Coalition forces caused 12% (*n* = 11,516); Anti-Coalition forces caused 11% (*n* = 9,954); and 2% of civilian deaths were from military crossfire where death could not be reliably attributed to a single perpetrator (*n* = 2,227). Of all years, the fourth postinvasion year (2006–2007) had the greatest number of civilian deaths from violence (*n* = 30,571). During the first postinvasion year (March 2003–March 2004), Coalition forces caused 52% of civilian deaths from armed violence (*n* = 7,252). In following years, the majority of civilians were killed by Unknown perpetrators (range *n* = 7,096 to 26,480 deaths per year, 61% to 87% of civilian deaths per year).

**Table 1:** Number (%) of 92,614 Iraqi civilian deaths by perpetrator and postinvasion year: From all armed violence, from short-duration violent events, and from long-duration violent events and aggregate reports.

Perpetrator	Reported Armed Violence Deathsa,b,c	5-y Total	Year 1	Year 2	Year 3	Year 4	Year 5
		3/20/03–3/19/08	3/20/03–3/19/04	3/20/04–3/19/05	3/20/05–3/19/06	3/20/06–3/19/07	3/20/07–3/19/08
**Unknown only** d	**All**	**68,396 (73.9)**	**5,655 (40.7)**	**7,096 (60.6)**	**12,868 (80.0)**	**26,480 (86.6)**	**16,297 (80.0)**
	*Short-duration events*	*44,750 (48.3)*	*780 (5.6)*	*1,911 (16.3)*	*6,072 (37.8)*	*20,354 (66.6)*	*15,633 (76.7)*
	*Long-duration and aggregate*	*23,646 (25.5)*	*4,875 (35.1)*	*5,185 (44.3)*	*6,796 (42.3)*	*6,126 (20.0)*	*664 (3.3)*
**Coalition only**	**All**	**11,516 (12.4)**	**7,252 (52.2)**	**2,175 (18.6)**	**530 (3.3)**	**693 (2.3)**	**866 (4.2)**
	*Short-duration events*	*3,990 (4.3)*	*1,415 (10.2)*	*802 (6.9)*	*362 (2.3)*	*604 (2.0)*	*807 (4.0)*
	*Long-duration and aggregate*	*7,526 (8.1)*	*5,837 (42.0)*	*1,373 (11.7)*	*168 (1.0)*	*89 (0.3)*	*59 (0.3)*
**Anti-Coalition only** e	**All**	**9,954 (10.7)**	**608 (4.4)**	**1,877 (16.0)**	**2,388 (14.9)**	**2,670 (8.7)**	**2,411 (11.8)**
	*Short-duration events*	*9,511 (10.3)*	*430 (3.1)*	*1,782 (15.2)*	*2,237 (13.9)*	*2,659 (8.7)*	*2,403 (11.8)*
	*Long-duration and aggregate*	*443 (0.5)*	*178 (1.3)*	*95 (0.8)*	*151 (0.9)*	*11 (0.0)*	*8 (0.0)*
**Crossfire, ≥2 Perpetrators**	**All**	**2,227 (2.4)**	**335 (2.4)**	**506 (4.3)**	**228 (1.4)**	**642 (2.1)**	**516 (2.5)**
	*Short-duration events*	*1,740 (1.9)*	*91 (0.7)*	*364 (3.1)*	*159 (1.0)*	*622 (2.0)*	*504 (2.5)*
	*Long-duration and aggregate*	*487 (0.5)*	*244 (1.8)*	*142 (1.2)*	*69 (0.4)*	*20 (0.0)*	*12 (0.0)*
**Other only** f	**All**	**521 (0.6)**	**37 (0.3)**	**46 (0.4)**	**60 (0.4)**	**86 (0.3)**	**292 (1.4)**
	*Short-duration events*	*490 (0.5)*	*32 (0.2)*	*46 (0.4)*	*42 (0.3)*	*83 (0.3)*	*287 (1.4)*
	*Long-duration and aggregate*	*31 (0.03)*	*5 (0.0)*	*0 (0)*	*18 (0.1)*	*3 (0.0)*	*5 (0.0)*
**Totals**	**All**	**92,614 (100.0)**	**13,887 (100.0)**	**11,700 (100.0)**	**16,074 (100.0)**	**30,571 (100.0)**	**20,382 (100.0)**
	*Short-duration events*	*60,481 (65.3)*	*2,748 (19.8)*	*4,905 (41.9)*	*8,872 (55.2)*	*24,322 (79.6)*	*19,634 (96.3)*
	*Long-duration and aggregate*	*32,133 (34.7)*	*11,139 (80.2)*	*6,795 (58.1)*	*7,202 (44.8)*	*6,249 (20.4)*	*748 (3.7)*

a Deaths from violent events of any duration that caused at least one reported civilian death and deaths from hospital and morgue aggregate reports (*n* = 92,614).

b Deaths from short-duration violent events lasting 2 d or less that caused at least one reported civilian death (*n* = 60,481).

c Deaths from long-duration violent events lasting over 2 d that caused at least one reported civilian death, and deaths from hospital and morgue aggregate reports (*n* = 32,133).

d Unknown perpetrators had a civilian target of attack, while themselves being indistinguishable from civilians (e.g., sectarian and Anti-Coalition combatants and criminals that attacked civilians).

e Anti-Coalition forces were identified by the presence of a Coalition-associated target of attack, as they generally did not wear uniforms or distinguishing marks in attacks.

f Primarily Iraqi police and Iraqi military forces.

Table 1 shows the origins of violent death data for perpetrators and years. We found that 65% of all deaths (*n* = 60,481/92,614) were documented from short-duration violent events that lasted 2 d or less. 35% of all deaths (*n* = 32,133/92,614) were documented from hospital and morgue aggregate reports or from long-duration violent events lasting over 2 d, such as the first battle of Fallujah (April–May 2004), the second battle of Fallujah (November–December 2004), and prolonged events during the invasion (March 20, 2003 to May 1, 2003).

### Perpetrators and Their Weapons: Civilian Deaths in Short-Duration Violence

We analyzed the effects of perpetrators and their weapons on civilians using the dataset of 60,481 Iraqi civilian deaths from 14,196 short-duration violent events that caused at least one reported civilian death. Reports on short-duration violent events link perpetrators using particular methods in a specific time and place to their resulting civilian deaths. Findings are of documented deaths of individuals; they are not estimates. Other than Table 1, all tables and figures describe deaths from short-duration violence only and exclude the 32,133 deaths from reports of prolonged violence lasting over 2 d and deaths reported only in aggregate summaries (predominantly morgue-reported deaths from executions and small arms gunfire).

**Figure 1:**
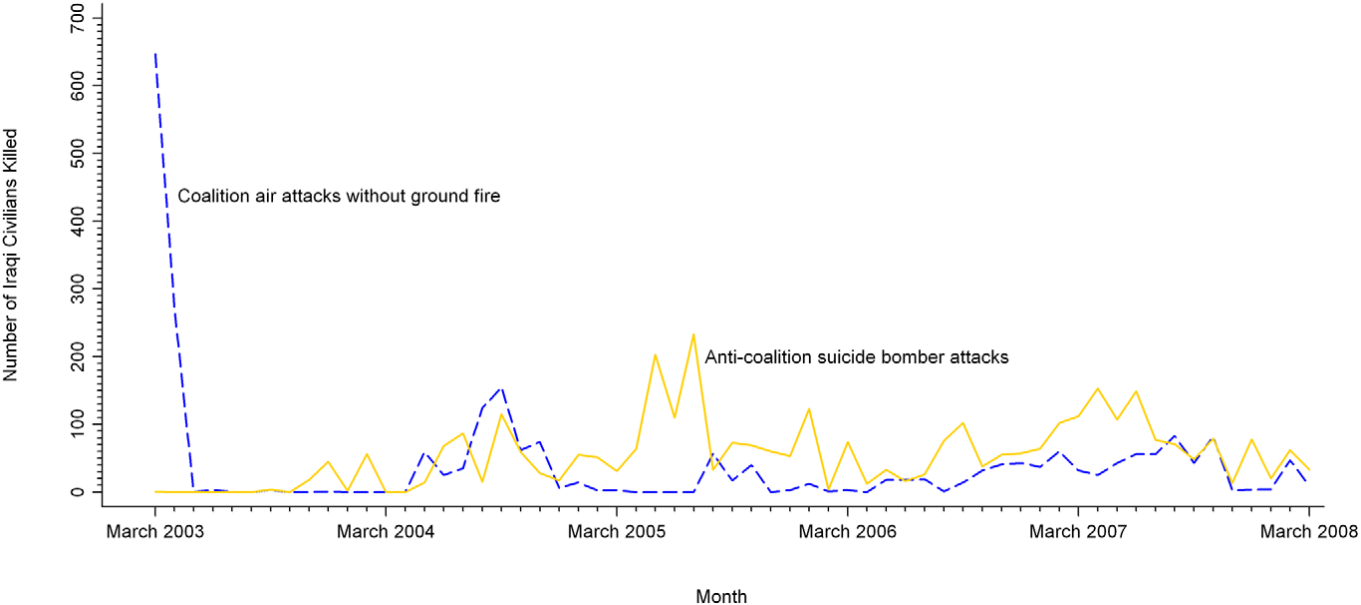
Monthly civilian deaths from Coalition air attacks and Anti-Coalition suicide bombers. Analysis of reported civilian deaths from short-duration violent events lasting 2 d or less, March 20, 2003 through March 19, 2008.

As shown in Table 2, one-third of deaths were in executions by Unknown perpetrators (*n* = 19,321/60,481, 32%). Unknown perpetrators killed close to one-half of their victims by execution (*n* = 19,321/44,750, 43%), leaving marks of torture on 29% of their executed victims (*n* = 5,697/19,321). Among methods used by Anti-Coalition forces, suicide bombers killed the greatest proportion of Anti-Coalition civilian victims (*n* = 3,333/9,511, 35%). Figure 1 shows Iraqi civilian deaths from Anti-Coalition suicide bombers over time: deaths peaked in 2005, with substantial deaths in 2004, and in mid-2006 to 2007. Among methods used by Coalition forces, air attacks without ground fire killed the greatest proportion of Coalition civilian victims (*n* = 2,384/3,990, 60%; Table 2). Figure 1 shows deaths from Coalition air attacks without ground fire over time: A high peak of Iraqi civilian deaths from Coalition air attacks occurred during the invasion in 2003, with lower peaks in 2004 and in mid-2006 to 2007.

**Table 2:** Iraqi civilian deaths from perpetrators using particular methods: analysis of 60,481 deaths from 14,196 events of short-duration armed violence, March 20, 2003 through March 19, 2008.

Methoda,b,c	Unknown Perpetrator Only				Anti-Coalition Perpetrator Only				Coalition Perpetrator Only			
	Civilian Deaths	Percent	Events	Mean (SE) Deaths/Event	Civilian Deaths	Percent	Events	Mean (SE) Deaths/Event	Civilian Deaths	Percent	Events	Mean (SE) Deaths/Event
**Execution, any** d	**19,321**	31.9	2,786	7 (0.2)	**316**	0.5	45	7 (1.2)	**54**	0.1	10	5 (2.2)
*Execution with torture* d	*5,697*	*9.4*	*704*	*8 (0.4)*	*60*	*0.1*	*9*	*7 (1.6)*	*0*	*0*	*0*	*0*
**Small arms gunfire** e	**8,086**	13.4	4,337	2 (0.03)	**1,526**	2.5	645	2 (0.1)	**987**	1.6	489	2 (0.1)
**Suicide bomb, any**	**5,363**	8.9	282	19 (2.3)	**3,333**	5.5	441	8 (0.5)	**0**	0	0	0
*Suicide bomber in vehicle*	*3,029*	*5.0*	*162*	*19 (3.7)*	*2,370*	*3.9*	*351*	*7 (0.5)*	*0*	*0*	*0*	*0*
*Suicide bomber on foot*	*2,320*	*3.8*	*119*	*19 (2.4)*	*963*	*1.6*	*90*	*11 (1.5)*	*0*	*0*	*0*	*0*
**Vehicle bomb**	**3,748**	6.2	541	7 (0.5)	**1,612**	2.7	325	5 (0.5)	**0**	0	0	0
**Roadside bomb**	**1,561**	2.6	712	2 (0.1)	**1,293**	2.1	692	2 (0.1)	**0**	0	0	0
**Mortar fire**	**1,763**	2.9	668	3 (0.1)	**289**	0.5	107	3 (0.2)	**19**	0.03	8	2 (0.6)
**Air attack without ground fire**	**0**	0	0	0	**0**	0	0	0	**2,384**	3.9	252	9 (0.9)
*Bombs only*	*0*	*0*	*0*	*0*	*0*	*0*	*0*	*0*	*479*	*0.8*	*28*	*17 (3.6)*
*Missiles only*	*0*	*0*	*0*	*0*	*0*	*0*	*0*	*0*	*353*	*0.6*	*43*	*8 (2.4)*
**Air attack with ground fire**	**0**	0	0	0	**0**	0	0	0	**213**	0.4	17	13 (3.2)
**Totals for single perpetrators, any method** f	**44,750**	74.0	10,211	4 (0.1)	**9,511**	15.7	2,517	4 (0.1)	**3,990**	6.6	893	4 (0.3)

a Deaths attributed to a single perpetrator group alone. Of the total 60,481 deaths, 2,230 deaths were attributed to “other” or “crossfire” and are not shown. Deaths were attributed to Unknown perpetrators if an unidentified perpetrator attacked a civilian target and to Anti-Coalition perpetrators if the target was Coalition or Coalition-associated.

b Short-duration events lasting 2 d or less that caused at least one reported civilian death.

c Unless noted, data are for events involving the single method used alone (e.g., small arms gunfire only, not events of combined gunfire and mortar fire).

d The extrajudicial killing of any captured individual by any method. Includes combatants extrajudicially executed postcapture, as after capture they become noncombatants protected under international humanitarian law [15],[16]. For executions only, “events” refer to events of discovering bodies, as events of killing by execution are usually hidden, and “mean” refers to number of bodies discovered.

e Open small arms gunfire, not including executions of captured individuals by gunfire.

f Included in Totals are deaths from events involving “other,” “unknown,” or “combined” methods if attributable to the single perpetrator, not shown in the single-method rows above.

SE, standard error.

An additional measure of the civilian impact of perpetrators' weapons is shown in Table 2: the mean (i.e., average) number of civilian deaths from a short-duration violent event where a perpetrator caused at least one civilian to die. The highest average number of civilian deaths per event occurred when Unknown perpetrators deployed suicide bombers against civilian targets (Unknown suicide bombers, both on foot and in a vehicle, killed 19 civilians per lethal event), and from Coalition aerial bombings (17 civilian deaths per lethal event, *p* = 0.8). Among Anti-Coalition methods, Anti-Coalition suicide bombers on foot killed the most Iraqi civilians per event (11 civilian deaths per lethal event), significantly more than Anti-Coalition suicide bombers in vehicles (seven civilian deaths per lethal event, *p*<0.01).

We compared mean numbers of civilians killed by lethal events from different modes of delivery of improvised explosive devices (IEDs) by comparing suicide bombs, vehicle bombs, and roadside bombs. Table 2 shows that for both Unknown perpetrators and Anti-Coalition perpetrators, suicide bomber IEDs killed the most Iraqi civilians per lethal event, nonsuicide vehicle-borne IEDs less, and roadside IEDs the least: Unknown perpetrators killed, on average, 19 civilians per suicide bomber IED event, seven per nonsuicide vehicle-borne IED event, and two per roadside IED event (*p*<0.0001). Anti-Coalition perpetrators killed, on average, eight civilians per suicide bomber IED event, five per nonsuicide vehicle-borne IED event, and two per roadside IED event (*p*<0.0001).

### Geographic Distribution of Violent Deaths, Executions, and Torture before Execution

Table 3 lists Iraq's 18 governorates with their total reported civilian deaths from short-duration violence, numbers, and proportions executed by Unknown perpetrators, and numbers and proportions whose bodies showed signs of torture before execution. Baghdad governorate had the highest number of civilian deaths from short-duration violence (*n* = 27,050), with 43% executed by Unknown perpetrators (*n* = 11,728/27,050) including 14% tortured before execution (*n* = 3,863/27,050). Wassit had a relatively low number of violent deaths (*n* = 1,503), but very high proportions of deaths by execution (57%) and torture before execution (21%). A factor in Wassit's unusually high proportion of reported executions is that the Tigris River flows from Baghdad through Wassit by the town of Swaira, where a system of weirs, originally designed to trap lily pads, catches corpses carried downstream from Baghdad [25]. Dahuk had a high proportion of executions (76%), but was based on a very small number of deaths (*n* = 19/25).

**Table 3:** Civilian deaths from short-duration violent events in Iraq's governorates.

Governorate	Deaths from Short-Duration Armed Violence	*n* (%) Executed by Unknown Perpetrators	*n* (%) Tortured before Execution by Unknown Perpetrators
Baghdad	27,050	11,728 (43)	3,863 (14)
Diyala	8,177	2,339 (29)	552 (7)
Ninewa	5,248	1,148 (22)	173 (3)
Anbar	4,222	995 (24)	294 (7)
Salah al-Din	4,134	778 (19)	105 (3)
Babylon	3,696	761 (21)	223 (6)
Tameem	2,019	306 (15)	103 (5)
Wassit	1,503	849 (57)	310 (21)
Basrah	1,173	181 (15)	47 (4)
Najaf	1,040	11 (1)	0 (0)
Kerbala	826	102 (12)	10 (1)
Qadissiya	396	68 (17)	14 (4)
Erbil	295	4 (1)	0 (0)
Missan	251	16 (6)	3 (1)
Sulaymaniyah	155	12 (8)	0 (0)
Thi-Qar	168	0 (0)	0 (0)
Muthanna	69	3 (4)	0 (0)
Unknown	34	1 (3)	0 (0)
Dahuk	25	19 (76)	0 (0)
Total	60,481	19,321 (32)	5,697 (9)

Reported civilian deaths from short-duration violent events lasting 2 d or less, March 20, 2003 through March 19, 2008.

**Figure 2:**
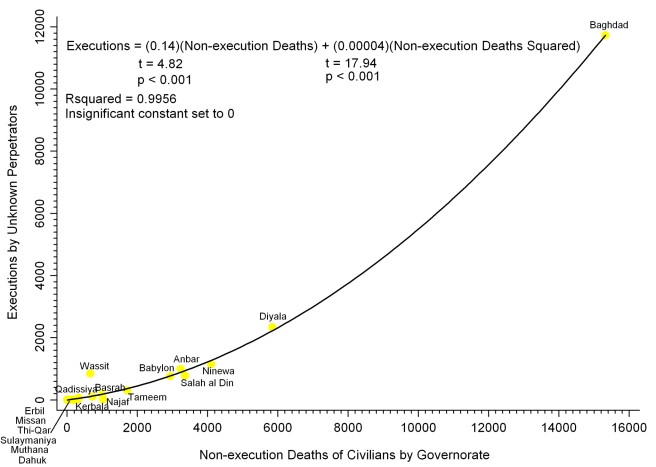
Iraqi governorates: nonexecution deaths versus executions by Unknown perpetrators. Analysis of reported civilian deaths from short-duration violent events lasting 2 d or less, March 20, 2003 through March 19, 2008.

Figure 2 shows the overall relationship between nonexecution violent deaths and deaths from Unknown perpetrators carrying out extrajudicial executions in Iraq's governorates. The statistically significant, increasingly steep curve of the quadratic regression indicates that as areas have higher numbers of nonexecution violent deaths of civilians, they have increasingly higher numbers of civilians executed by Unknown perpetrators. To illustrate: if starting with 1,000 nonexecution deaths, an additional 100 nonexecution deaths predicts an additional 22.4 execution deaths, whereas if starting with 2,000 nonexecution deaths, an additional 100 nonexecution deaths predicts an additional 30.4 execution deaths, with increasingly higher rates of execution deaths occurring with higher numbers of nonexecution deaths. A quadratic regression for torture before execution in Iraqi governorates (unpublished data) similarly indicates that as governorates have higher numbers of nonexecution violent deaths of civilians, they have increasingly higher numbers of civilians tortured before execution by Unknown perpetrators (executions  = (0.00002) (nonexecution deaths squared), *t* = 43.99, *R^2^* = 0.9913, *p*<0.001).

### Perpetrators and Victim Demographics

On the basis of demographic information in reports, we identified the 60,481 civilian victims of short-duration violence to be: 17,939 (29.7%) men; 1,981 (3.3%) women; 1,515 (2.5%) adults of unreported sex; 2,146 (3.5%) children under age 18; and 36,900 (61.0%) civilian victims of unreported age. Among the 22,066 Iraqi civilian victims demographically identifiable as men, women, or children, 81.3% were men (*n* = 17,939/22,066), 9.0% were women (*n* = 1,981/22,066), and 9.7% were children (*n* = 2,146/22,066). Because our earlier analysis of this dataset applied the category of “female” rather than “woman” [10], we compared these categorizations: Female deaths (*n* = 2,396) consisted of 83% women (*n* = 1,981), 12% girls under age 18 (*n* = 299), and 5% females of unknown age (*n* = 116), indicating substantial generalizability between findings for females and women. Among 2,146 child deaths, 559 (26.0%) were identified as boys, 299 (13.9%) as girls, and 1,288 (60.0%) were children of unreported sex.

Table 4 shows victim demographics from perpetrators and their specific methods of short-duration armed violence for 60,481 Iraqi civilian deaths. Unknown perpetrators targeting civilians caused 66.9% of all violent deaths of civilian men (*n* = 12,007/17,939), mostly in executions (32.2%, *n* = 5,768/17,939); 68.8% of all violent deaths of women (*n* = 1,363/1,981), mostly by gunfire (21.1%, *n* = 418/1,981); and 52.2% of all violent deaths of children (*n* = 1,120/2,146), again mostly by gunfire (9.8%, *n* = 211/2,146). Anti-Coalition forces caused 25.8% of violent deaths of civilian men (*n* = 4,629/17,939); 8.7% of violent deaths of women (*n* = 173/1,981); and 16.5% of violent deaths of children (*n* = 355/2,146), in all cases mostly by suicide bombing (7.9%, 4.0%, and 7.0%, respectively). More women and children were killed by Anti-Coalition suicide bombers in vehicles (*n* = 210) than on foot (*n* = 21). Coalition forces caused 4.1% of violent deaths of civilian men (*n* = 741/17,939), mostly by gunfire (2.2%, *n* = 390/17,939); 15.0% of violent deaths of women (*n* = 297/1,981), mostly by air attacks without ground fire (9.7%, *n* = 193/1,981); and 21.8% of violent deaths of children (*n* = 467/2,146), again mostly by air attacks without ground fire (13.2%, *n* = 284/2,146). For the 36,900 civilian victims of unreported age, 78.8% were killed by Unknown perpetrators (*n* = 29,064/36,900), mostly in executions (35.2%, *n* = 12,988/36,900); 11.2% by Anti-Coalition forces (*n* = 4,126/36,900), mostly by suicide bombing (4.5%, *n* = 1,656/36,900); and 6.6% by Coalition forces (*n* = 2,438/36,900), mostly by air attacks without ground fire (4.5%, *n* = 1,674/36,900).

**Table 4:** Victim demographics and DWI outcomes from perpetrators using particular methods: analysis of 60,481 Iraqi civilian deaths from short-duration violence, March 20, 2003 through March 19, 2008.

Methoda,b,c	Unknown Perpetrator Only					Anti-Coalition Perpetrator Only					Coalition Perpetrator Only				
	Men (%)^d^	Women (%)^e^	Children (%)^f^ [Boys + Girls + CUS]	Age Unreported (%)^g^	Woman and Child DWI^h^	Men (%)^d^	Women (%)^e^	Children (%)^f^ [Boys + Girls + CUS]	Age Unreported (%)^g^	Woman and Child DWI^h^	Men (%)^d^	Women (%)^e^	Children (%)^f^ [Boys + Girls + CUS]	Age Unreported (%)^g^	Woman and Child DWI^h^
**Execution, any**	5,768 (32.2)	260 (13.1)	115 (5.4) [64+8+43]	12,988 (35.2)	**6**	297 (1.7)	2 (0.1)	0	16 (0.0)	**1**	24 (0.1)	6 (0.3)	9 (0.4) [2+7+0]	15 (0.0)	**38**
*Execution with torture*	*1,735 (9.7)*	*49 (2.5)*	*16 (0.7) [12+0+4]*	*3,878 (10.5)*	***4***	*60 (0.3)*	*0*	*0*	*0*	***0***	*0*	*0*	*0*	*0*	*0*
**Small arms gunfire**	4,159 (23.2)	418 (21.1)	211 (9.8) [70+44+97]	2,609 (7.1)	**13**	1,246 (6.9)	14 (0.7)	11 (0.5) [4+1+6]	160 (0.4)	**2**	390 (2.2)	67 (3.4)	111 (5.2) [41+19+51]	405 (1.1)	**31**
**Suicide bomb, any**	677 (3.8)	131 (6.6)	183 (8.5) [52+12+119]	4,318 (11.7)	**32**	1,416 (7.9)	80 (4.0)	151 (7.0) [38+12+101]	1,656 (4.5)	**14**	0	0	0	0	**0**
*Suicide bomber in vehicle*	*324 (1.8)*	*54 (2.7)*	*90 (4.2) [28+4+58]*	*2,559 (6.9)*	***31***	*901 (5.0)*	*66 (3.3)*	*144 (6.7) [36+10+98]*	*1,235 (3.3)*	***19***	*0*	*0*	*0*	*0*	*0*
*Suicide bomber on foot*	*344 (1.9)*	*77 (3.9)*	*93 (4.3) [24+8+61]*	*1,754 (4.8)*	***33***	*515 (2.9)*	*14 (0.7)*	*7 (0.3) [2+2+3]*	*421 (1.1)*	***4***	*0*	*0*	*0*	*0*	*0*
**Vehicle bomb**	264 (1.5)	177 (8.9)	137 (6.4) [21+8+108]	3,131 (8.5)	**54**	309 (1.7)	32 (1.6)	79 (3.7) [14+21+44]	1,176 (3.2)	**26**	0	0	0	0	**0**
**Roadside bomb**	345 (1.9)	78 (3.9)	108 (5.0) [24+21+63]	969 (2.6)	**35**	715 (4.0)	19 (1.0)	41 (1.9) [11+4+26]	476 (1.3)	**8**	0	0	0	0	**0**
**Mortar fire**	81 (0.5)	118 (6.0)	178 (8.3) [28+28+122]	1,361 (3.7)	**79**	85 (0.5)	9 (0.5)	46 (2.1) [12+9+25]	145 (0.4)	**39**	1 (0.0)	1 (0.1)	5 (0.2) [2+3+0]	12 (0.0)	**86**
**Air attack without ground fire, any**	0	0	0	0	**0**	0	0	0	0	**0**	210 (1.2)	193 (9.7)	284 (13.2) [56+41+187]	1,674 (4.5)	**69**
*Bombs only*	*0*	*0*	*0*	*0*	***0***	*0*	*0*	*0*	*0*	***0***	*22 (0.1)*	*14 (0.7)*	*34 (1.6) [11+6+17]*	*391 (1.2)*	*69*
*Missiles only*	*0*	*0*	*0*	*0*	***0***	*0*	*0*	*0*	*0*	***0***	*62 (0.3)*	*20 (1.0)*	*35 (1.6) [10+8+17]*	*236 (0.6)*	*47*
**Air attack with ground fire**	0	0	0	0	**0**	0	0	0	0	**0**	50 (0.3)	13 (0.7)	13 (0.6) [0+0+13]	136 (0.4)	**34**
**Totals for single perpetrators, any method** i	12,007 (66.9)	1,363 (68.8)	1,120 (52.2) [311+149+660]	29,064 (78.8)	**17**	4,629 (25.8)	173 (8.7)	355 (16.5) [83+47+225]	4,126 (11.2)	**10**	741 (4.1)	297 (15.0)	467 (21.8) [112+86+269]	2,438 (6.6)	**51**

a Of the total 60,481 deaths from short-duration violence, this Table shows 56,780 deaths attributed to these single perpetrator groups alone. Because of space constraints, we do not show the distribution of the 1,471 adult civilians of unreported sex attributed to “unknown,” “Anti-Coalition.” and “Coalition,” or the 2,230 deaths attributed to “other” or “crossfire” (which include *n* = 44 adult civilians of unreported sex from “other” or “crossfire” for the total of *n* = 1,515 adult civilians of unreported sex). Deaths were attributed to Unknown perpetrators if an unidentified perpetrator attacked a civilian target and to Anti-Coalition perpetrators if the target was Coalition or Coalition-associated.

b Short-duration events lasting 2 d or less that caused at least one reported civilian death.

c Unless noted, data are for events involving the single method used alone (e.g., small arms gunfire only, not combined gunfire and mortar fire).

d Number (%) of men among the total of 17,939 men civilian victims from all perpetrators and all methods.

e Number (%) of women among the total of 1,981 women civilian victims from all perpetrators and all methods.

f Number (%) of children among the total of 2,146 child victims from all perpetrators and all methods. Within [ ] are shown numbers of boys, girls, and children of unreported sex.

g Number (%) of unreported age among the total of 36,900 civilian victims of unreported age from all perpetrators and all methods.

h “Woman and Child DWI”  =  (*n* women + children killed/*n* women + children + men civilians killed) × (100). Possible DWI range is 0 to 100 [15]. Higher Woman and Child DWI outcomes indicate higher proportions of women and children among demographically identified civilian victims.

i Included in Totals are deaths from “other,” “unknown,” or “combined” methods if attributable to the single perpetrator, not shown in the single-method rows above.

CUS, children of unreported sex.

**Figure 3:**
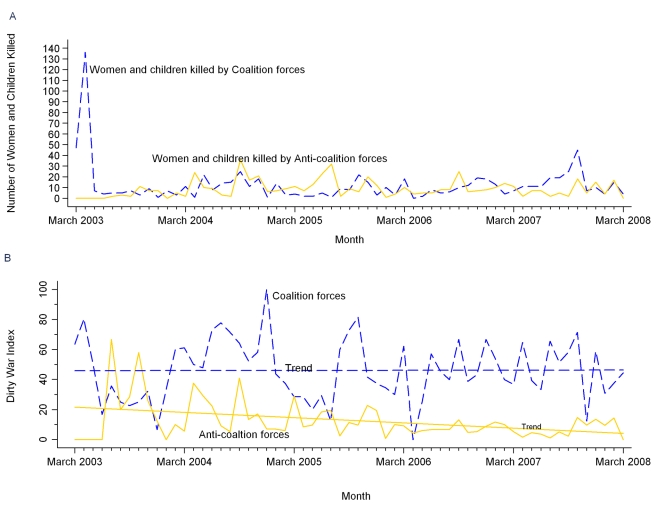
Violent deaths of Iraqi women and children by Coalition and Anti-Coalition forces. Analysis of reported civilian deaths from short-duration violent events lasting 2 d or less, March 20, 2003 through March 19, 2008. (A) Monthly numbers of women and children killed. (B) Monthly Woman and Child Dirty War Index.

A comparison of Coalition and Anti-Coalition effects on Iraqi women and children over time is made in Figure 3A, which shows monthly raw numbers of women and children killed in short-duration violence by Coalition forces (*n* = 764) and Anti-Coalition forces (*n* = 528). Woman and child deaths from Coalition forces peaked during the invasion in 2003, whereas those from Anti-Coalition forces peaked in 2004–2005.

Another comparison of perpetrators' civilian impact, made in Table 4, uses the Woman and Child DWI to indicate the proportion of a perpetrator's victims who were women or children among their civilian victims of known demographic status (possible DWI range 0–100) [15]. Differences between the proportion of women and children among civilians killed by perpetrators using each method were statistically significant with *p*<0.001, except between Unknown and Anti-Coalition perpetrators using execution with torture (DWI  = 4 versus DWI  = 0, *p* = 0.13). The overall Woman and Child DWI for all perpetrators and methods in Iraq combined during 2003–2008 was 18.7. For particular perpetrators and their methods, Woman and Child DWIs suggest that the “dirtiest” effects in terms of causing the highest rates of woman and child death were from Unknown perpetrator mortar fire (DWI  = 79), from Coalition air attacks without ground fire (DWI  = 69), and from Unknown perpetrator nonsuicide vehicle bombs (DWI  = 54), for cases where at least 50 civilian deaths were reported from a perpetrator's method.

Comparing civilian effects of opposing combatant forces, the total Anti-Coalition Woman and Child DWI of 10 and the total Coalition Woman and Child DWI of 51 suggest that Coalition forces caused a significantly higher proportion of woman and child deaths among its civilian deaths during 2003–2008 than did Anti-Coalition forces (*p*<0.001). In Figure 3B, Coalition and Anti-Coalition Woman and Child DWIs are shown over time. Linear regression of Coalition monthly Woman and Child DWIs shows that proportions of women and children among Coalition civilian victims did not change significantly over 2003–2008 (slope coefficient  = 0.0068, *t* = 0.05, *R^2^* = 0, *p* = 0.964). Linear regression of Anti-Coalition monthly Woman and Child DWIs shows a statistically significant downward trend in their Woman and Child DWIs over 2003–2008 (slope coefficient  = −0.2894, *t* = −3.20, *R^2^* = 0.15, *p* = 0.002).

### Small Arms Gunfire

**Figure 4:**
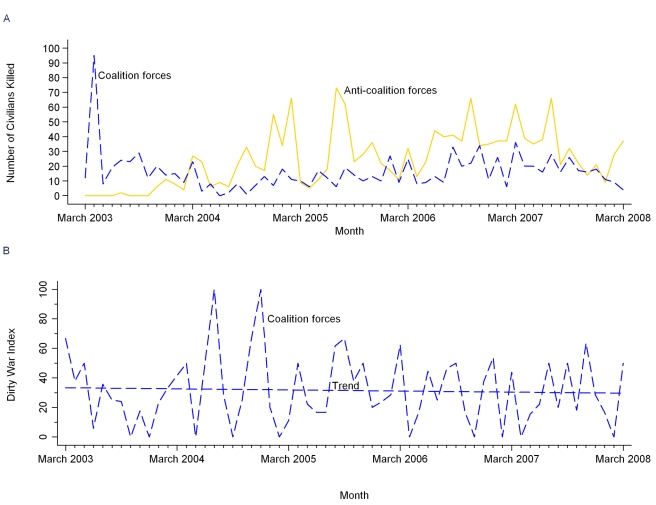
Civilian violent deaths from Coalition and Anti-Coalition small arms gunfire. Analysis of reported civilian deaths from short-duration violent events lasting 2 d or less, March 20, 2003 through March 19, 2008. (A) Monthly numbers of civilians killed by Coalition and Anti-Coalition gunfire. (B) Monthly Woman and Child Dirty War Index from Coalition gunfire.

Small arms firearms are designed to be carried by one individual and include handguns, automatic weapons, assault rifles, and machine guns [26]. Our data show that Unknown perpetrators caused the most small arms deaths of Iraqi civilians (*n* = 8,086; Table 2) and of women and children (*n* = 629; Table 4). In contrast to Unknown perpetrators, who directly targeted civilians, our data on civilian deaths from Anti-Coalition and Coalition small arms were generally from attacks on military targets, which permits direct comparison of the incidental (sometimes described as “collateral”) lethal effects on civilians when these opposing forces used this common weapon-type in Iraq's conflict environment. As shown in Table 2, both Anti-Coalition and Coalition forces killed an average of two Iraqi civilians per short-duration event where their gunfire caused any civilian death. In Figure 4A, which shows deaths over time, Iraqi civilian deaths from Anti-Coalition gunfire (*n* = 1,526) peaked in 2005, with another high, sustained peak from mid-2006 to mid-2007. Civilian deaths from Coalition gunfire (*n* = 987) peaked during the invasion, with a lower, more sustained peak from mid-2006 to mid-2007.

Demographic analysis of Coalition and Anti-Coalition small arms civilian deaths (Table 4) indicates that Coalition gunfire caused more woman and child deaths in raw numbers (*n* = 178 versus *n* = 25), and caused a significantly higher proportion of woman and child deaths among civilian gunfire victims (DWI  = 31 versus DWI  = 2, *p*<0.001). Figure 4B shows Coalition monthly Woman and Child DWIs from small arms gunfire over 2003–2008. Linear regression of monthly Coalition DWIs suggests that there was no statistically significant change in Woman and Child DWIs from Coalition gunfire over the first 5 y of the war (slope coefficient  = −0.0617, *t* = −0.35, *R^2^* = 0.002, *p* = 0.73).

## Discussion

Conflict-associated violent mortality is a product of perpetrators' behavior, their weapons technology, interactions between opponents, and context [27]–[29]. Our analysis shows how Iraqi civilian deaths from perpetrators of violence during the first 5 y of the Iraq war have varied over time, by geographic locale, according to perpetrators' weapons of choice, in demographic characteristics, and according to whether or not civilians are targeted.

### Civilian Deaths from Perpetrators

Three-quarters of Iraqi civilian victims of armed violence were killed in direct targeting, either by sectarian or Anti-Coalition combatants disguised as civilians, or by criminals (encompassed in our Unknown perpetrator category). When any military combatant intentionally targets civilians, this constitutes a war crime [15],[16],[19],[30]. Although a military force incurs primary responsibility for its civilian victims, whether intended or unintended, a possible factor affecting our comparison of civilian deaths from Anti-Coalition forces and Coalition forces is that Coalition combatants present clear, uniformed military targets to Anti-Coalition forces and are easily distinguished from Iraqi civilians. This would decrease the Anti-Coalition likelihood of killing civilians accidently. In contrast, Anti-Coalition combatants are visually indistinguishable from civilians during military actions and often fight from among civilians; practices that violate laws of war, displace their own risk as combatants onto Iraqi civilians, and predictably contribute to civilian deaths [15],[19],[30]–[32].

### Perpetrators and Explosive Weapons

Recent reports have emphasized the potential high risk to civilians from explosive weapons [5],[8],[33]. This concern is supported by our findings for Iraq. For events that caused a civilian death, the greatest average numbers of civilian deaths per event resulted from Unknown perpetrator suicide bombings and from Coalition air attacks. Civilian deaths from air attacks, which typically involve bombs or missiles, peaked during the invasion. Of all methods used by Unknown and Anti-Coalition perpetrators, suicide bombers killed the greatest numbers of Iraqi civilians per event. Unknown perpetrator suicide bombers directed against “soft,” civilian targets killed 11 more Iraqi civilians per event than Anti-Coalition suicide bombers directed against “hard,” Coalition targets (19 versus 8, *p*<0.001). Our findings support descriptions by others of suicide attacks as the “mass destruction” weapon of sectarian and Anti-Coalition forces in Iraq [17],[21],[22],[34],[35] and as the most lethal terrorist method internationally [34].

### Extrajudicial Executions

Execution by Unknown perpetrators was the most prevalent form of violent death affecting Iraqi civilians in 2003–2008. Although Unknown perpetrators' motivations cannot be ascertained from our data, our findings are compatible with descriptive reports of Iraq's postinvasion environment in this period, during which civilians were extensively abducted, ransomed, exchanged, and executed for financial or political gain, to destabilize Iraqi society, or to punish or deter “collaborators,” by perpetrators who strategically remained unidentifiable and who included a mixture of criminals, and sectarian and Anti-Coalition combatants, including within Iraqi security forces and police [17],[19],[21]–[24],[35]–[37].

Our findings on the geographic distribution and nature of violent death across Iraq's governorates show that deaths from executions, and executions with torture, progressively and disproportionately increased as deaths from other forms of violence increased. Further quantitative and qualitative study is needed to identify reasons behind this finding. We speculate that increased numbers of civilian violent deaths in governorates may indicate environments of lower security and greater impunity, which allow perpetrators to increase the scale of systematic use of executions—a cheap, low-technology form of armed violence—for purposes of retribution, punishment, intimidation, and financial gain. In Iraq, torture before execution and methods of disposal of corpses often leave mutilated bodies to be discovered [24],[25],[38]. In this way, perpetrators in Iraq may use bodies of tortured victims to send a message, to terrorize, to clear territory, to display the cost of resisting their power, to destabilize security [17],[24], or to increase the stakes in future ransom of abductees. On an individual level, increased exposure to violence, which can cause desensitization and peer-induced escalation [29], may disproportionately increase numbers of individuals willing to perpetrate executions or the propensity of perpetrators to inflict torture on captives.

### Perpetrators' Effects on Men, Women, and Children

Our demographic analysis shows that Iraqi civilian men are the main civilian victims of lethal armed violence in this war, as in other wars [17],[18], despite having the same protected civilian status as women and children civilians under laws of war [16],[39]. Because women and children are less targeted in Iraq's conflict, we use a Woman and Child DWI measuring the proportion of women and children among civilian victims of known demographic status to indicate relatively indiscriminate weapons or weapons-use in comparisons between perpetrators. Indiscriminate weapons and indiscriminate use of weapons are prohibited under laws of war [5],[8],[15],[16]. Although our Woman and Child DWI findings are not direct assessments of the legality of military actions under laws of war, and are subject to limitations discussed below, they can be useful to signal relatively higher-risk indiscriminate effects of perpetrator's weapons on women and children. We found the highest risks for indiscriminate effects on women and children when civilians were killed were from: Unknown perpetrators using mortars against civilians, Unknown perpetrators using nonsuicide vehicle bombs against civilians, and Coalition air attacks without ground fire, involving bombs or missiles. Compared to Anti-Coalition forces, Coalition forces caused a higher total Woman and Child DWI for 2003–2008, with no evidence of a significant decrease over time. Face validity of our findings on the Woman and Child DWI for Iraq's conflict environment is suggested by demographic data recently released by the Government of Iraq on 4,068 civilian violent deaths in 2009: 3,267 men, 439 women, and 362 children [40]. The Woman and Child DWI generated from Government of Iraq data for 2009 is 19.7 (DWI  = 439+362/439+362+3,267×100), which does not differ significantly from our overall Woman and Child DWI of 18.7 for 2003–2008 (*p* = 0.14).

Our temporal analysis of Coalition weapon-effects showed that numbers of woman and child deaths, and numbers of civilian deaths from air attacks, peaked during the invasion of March 20, 2003 to May 1, 2003, when the Coalition used heavy air power. Our findings are consistent with the 2004 Iraq Living Conditions Survey (ILCS), which found that of Iraqis chronically disabled from war injuries inflicted in the first year of this war, half of disabled adults were women, and over 15% of the disabled were children under age 10—unusually high proportions—with the civilian population most affected [41]. These findings, combined with the high Woman and Child DWI outcomes from air attacks, suggest that heavy reliance on air power during the invasion may have been particularly costly for Iraqi civilians—and especially for women and children—in terms of deaths and injuries. Our findings support the view that indiscriminate lethal effects of explosive aerial weapons on civilians need to be addressed through changed practice and policy on the use of air power in armed conflict, with air attacks on populated areas prohibited or systematically monitored to demonstrate civilian protection [5],[8],[10],[33].

### Small Arms Gunfire

Most civilian deaths from small arms gunfire were caused by Unknown perpetrators targeting civilians. Although Anti-Coalition forces and Coalition forces caused the same average number of civilian gunfire deaths per lethal event while firing on presumably military targets, our findings suggest that Coalition gunfire had a more indiscriminate effect on Iraqi women and children. A possible factor may be that Anti-Coalition forces often wage battle amid or near civilians, sometimes from homes with women and children, thereby placing these civilians at risk of Coalition fire [30],[31]. Also, we counted among Anti-Coalition civilian victims Iraqi police who were killed while in civil roles at Coalition-associated targets such as police stations, academies or checkpoints. Male dominance of this targeted profession may have lowered Anti-Coalition Woman and Child DWIs from small arms, and from other Anti-Coalition weapons. Possible contributing Coalition factors include reports of indiscriminate or disproportionate Coalition gunfire during raids on Iraqi homes [30],[42], into urban residential surroundings [30],[42], and near convoys and roadside checkpoints [17],[23],[30],[42],[43] when perceiving threat, when attacked and after being attacked [17],[23],[30]. It has been suggested that Coalition troops may have fired more indiscriminately when shifted rapidly between roles of active combat and civilian engagement, and when in conditions of inadequate training and interpreters, poorly marked checkpoints, or low accountability [30],[42],[43]. Although it has been reported that these problems were addressed [30],[43] and that civilian deaths from Coalition gunfire decreased as a result [30], our data show no evidence of a significant decrease in numbers of civilian deaths from Coalition gunfire during the period of our study, or in Woman and Child DWIs. Our findings suggest that relatively indiscriminate effects from Coalition gunfire persisted over 5 y postinvasion, and that military efforts to minimize civilian casualties need to be coupled with systematic monitoring of casualties in order to assess and strengthen civilian protection.

### Limitations

We do not examine indirect deaths from war, deaths of combatants, or perpetrators' patterns of civilian deaths after the first 5 y of the war. Although it is not possible for this paper to describe detailed, event-based demographic, temporal, and geographic patterns of civilian deaths from perpetrators and their weapons that occurred after our study's 5-y time-period (March 20, 2003 to March 19, 2008), we can provide an overview based on the public database of the IBC Web site [13]: Monthly rates of Iraqi civilian deaths from armed violence averaged 1,518 deaths per month for the period of our study, then began to decline in May of 2008. Since July 2008, deaths have persistently varied between 200 to under 600 per month, averaging 401 deaths per month. This paper's analysis of 92,614 civilian deaths from armed violence covers 86% of all 108,107 Iraqi civilian deaths from armed violence recorded by IBC from the beginning of the Iraq war to the latest available date in November 2010. Our findings do not represent total violent deaths or events affecting Iraqi civilians, as not every death or event are reported. For deaths from perpetrators' weapon-types, we only analyzed those attributable to single weapon-types and single perpetrators in short-duration events. This analysis understates total absolute numbers killed by each perpetrator's weapon-type, but increases reliability in attributing deaths and allows comparison of proportional effects within a uniform time-frame.

In attributing civilian deaths to perpetrators, Coalition forces are generally identifiable, so Coalition-induced civilian deaths are directly measured. In contrast, Anti-Coalition forces are indirectly identified by their Coalition target; a necessarily conservative identification of Anti-Coalition perpetrators for accuracy because Anti-Coalition forces routinely avoid identification by uniforms or markings. Anti-Coalition-caused deaths identified in this dataset therefore only represent civilians killed by Anti-Coalition military targeting. An unknown, additional number were killed in direct civilian targeting by unidentifiable Anti-Coalition forces, included within our category of Unknown perpetrators, whose findings as a whole can be understood to represent direct civilian targeting. A factor that increases child deaths from Coalition forces is the use by Anti-Coalition forces of children age 10 to 17 in attacks against Coalition forces [44]–[46]. Use of child soldiers is against laws of war and a war crime if the child is under age 15 [15],[16]. Although quantitative data are very limited [44]–[46], child soldiers are not prevalent in Iraq's conflict. However, some child deaths in our study are likely to be of children placed in combatant roles at their time of death.

Although we know of no evidence that media coverage bias affects armed conflict reporting on civilian victim sex or age, we consider it possible that media reports may identify women and children more readily than men civilians among the dead, perhaps for human interest or from a normative assumption that a victim of armed violence is a man unless stated otherwise. If this bias exists it could inflate woman and child percentage findings among civilian deaths generally for all perpetrators and weapons. Indirect evidence against this bias is the similar proportions of women and children among violent deaths of civilian men, women, and children found from our IBC data for 2003–2008 and from Government of Iraq data for 2009 (DWI  = 18.7% versus DWI  = 19.7%, respectively) [40]. (Government data on victim demographics are unavailable for 2003–2008.) However, until more directly comparable data are available for replication, we suggest that our Woman and Child DWI findings should be considered robust indicators of the relative demographic characteristics of civilian deaths, rather than absolute proportions of civilian deaths. There is no evidence to suggest that differential reporting bias between perpetrators or weapons affects our comparisons of mean numbers of civilians killed per event or of proportions of women and children among civilian deaths (e.g., that woman and child deaths are more likely to be reported than men for one perpetrator than another, or for one weapon than another). However, our Woman and Child DWI findings should be considered suggestive until careful studies for possible reporting bias are done. Although we show in Table 4 unreported demographic data for readers' information (civilian victims of unreported age, children of unreported sex, and in the footnote, adults of unreported sex), assessment for bias in reporting demographic information requires knowing the true demographic composition of the unknowns within this dataset, or comparison to a matched, independent dataset of comparable detail. A study using the IBC database found no media-reporting bias for governorates with higher levels of insurgent violence to have any more, or less, missing information on event location, but missing demographic information was not examined [47]. It has been established that media reports can provide systematic, meaningful data on conflict casualties [1],[7],[10],[11],[34],[48]–[50]. A general limitation of conflict studies that use media reports is that journalists collect information in their reports for purposes other than systematic inquiry and, as we illustrate here, study is needed to assess possible bias in media reports describing perpetrators, weapons, and victims of armed conflict. Establishing standards for reporting victim information could maximize the contribution of media reports to understanding violence.

A strength of our study is its use of verifiable data on 92,614 actual civilian deaths from armed violence. Surveys extrapolate from relatively few actual violent deaths, e.g., 164 violent deaths in the Iraq Family Health Survey (IFHS) of 9,345 households [51], with few deaths traceable to specific weapons or events. Although epidemiological surveys in armed conflicts can provide good mortality data, they can be affected by recall bias, reporting bias, survival bias, and difficulties in implementation [3],[47],[52],[53]. IBC's monitoring using daily-collated data minimizes recall bias (99% of events were investigated and reported within 24 h [13]) and permits surveillance over time of traceable events—valuable attributes for monitoring and analyzing conflict mortality trends [3],[7],[48],[51]. IBC data correlate with IFHS data showing similar trends and distribution of violent deaths by governorate [51] and with ILCS data [41] for war-related deaths by governorate [54], with some differences because the surveys did not always differentiate combatant from civilian deaths, as is often the case in surveys owing to sensitivity, danger, or response unreliability [52],[55],[56]. Clinical studies can provide data on civilian conflict mortality from weapon-types. However, most clinical studies use aggregate hospital samples untraceable to causative events or perpetrators, and results can be biased by prehospital patterns of mortality that vary by weapon or injury, and by local treatment access and quality [57]–[61].

Our analysis describes only violence that resulted in a civilian death. As of the time of writing this paper, military actors have not released systematic data on their use of weapons in events that leave civilians unharmed; data that would allow analysis of their practice of civilian-protective warfare. Anti-Coalition forces release no data on use of weapons, and among Coalition forces, the US Air Force has released only partial data on some types of air strikes and munitions dropped in Iraq, without systematic reports of actions that caused or avoided civilian casualties [62]–[65]. The recent, unauthorized release of US Army SIGACT (significant activity) records covering 2004–2009 by WikiLeaks will yield data on weapons, perpetrators, and casualties from the perspective of the US military, but will require time for careful analysis [66]. Use of transparent, verifiable systems for tracking and measuring civilian and combatant casualties from all military actions could identify civilian-protective tactics (e.g., careful targeting, or less frequent use of weapons) and would allow rapid identification, correction, and deterrence of civilian-harmful tactics (e.g., use of indiscriminate weapons near civilians) [8],[15],[67]. Improved civilian protection decreases preventable civilian deaths, physical injuries, and psychological injuries, such as the increased mental disorders found in Iraqi citizens exposed to bomb blasts, mutilated bodies, and gunfire [68]. Our findings on civilian deaths from perpetrators and their weapons during 5 y of the Iraq war illustrate the feasibility as well as the public health and humanitarian potential of detailed tracking of war's effects on a civilian population.

## Abbreviations

DWI: Dirty War Index
IBC: Iraq Body Count
IED: improvised explosive device

## References

[1] Coupland R (2007). Security, insecurity and health.. Bull World Health Organ.

[2] Krug EG, Dahlberg LL, Mercy JA, Zwi AB, Lozano R (2002). World report on violence and health..

[3] Murray CJL, King G, Lopez AD, Tomijima N, Krug EG (2002). Armed conflict as a public health problem.. BMJ.

[4] Tam CC, Lopman BA, Bornemisza O, Sondorp E (2004). Epidemiology in conflict – a call to arms.. Emerg Themes Epidemiol.

[5] Landmine Action (2009). Explosive violence: the problem of explosive weapons..

[6] Pedersen J (2009). Health and conflict: a review of the links..

[7] Geneva Declaration Secretariat (2008). Global burden of armed violence..

[8] United Nations Institute for Disarmament Research (2010). Explosive weapons: framing the problem. Background paper number 1 of the Discourse on Explosive Weapons (DEW) project, April 2010.. http://explosiveweapons.info/category/unidir/.

[9] Levy BS, Sidel VW (2008). War and public health. 2nd edition..

[10] Hicks MH, Dardagan H, Guerrero Serdán G, Bagnall PM, Sloboda JA (2009). The weapons that kill civilians — deaths of children and noncombatants in Iraq, 2003-2008.. N Engl J Med.

[11] Taback N, Coupland R (2005). Towards collation and modeling of the global cost of armed violence on civilians.. Med Confl Surviv.

[12] Verwimp P (2006). Machetes and firearms: the organization of massacres in Rwanda.. J Peace Res.

[13] Iraq Body Count.. http://www.iraqbodycount.org.

[14] Dardagan H, Sloboda J, Williams K, Bagnall P (2005). Iraq Body Count: a dossier of civilian casualties 2003-2005..

[15] Hicks MH, Spagat M (2008). The Dirty War Index: a public health and human rights tool for examining and monitoring armed conflict outcomes.. PLoS Med.

[16] International Committee of the Red Cross (2010). International humanitarian law.. http://www.icrc.org/eng/ihl.

[17] Slim H (2007). Killing civilians: method, madness and morality in war..

[18] Carpenter RC (2006). Recognizing gender-based violence against civilian men and boys in conflict situations.. Security Dialogue.

[19] Human Rights Watch (2005). A face and a name: civilian victims of insurgent groups in Iraq. 17(9).. http://www.hrw.org/en/reports/2005/10/02/face-and-name.

[20] Convention on the Rights of the Child, (1989). Geneva: UN Office of the High Commissioner for Human Rights.. http://www2.ohchr.org/english/law/crc.htm.

[21] Hafez MM (2006). Suicide terrorism in Iraq: a preliminary assessment of the quantitative data and documentary evidence.. Stud Confl Terror.

[22] Hashim AS (2006). Insurgency and counter-insurgency in Iraq..

[23] Chehab Z (2006). Iraq ablaze: inside the insurgency..

[24] Green P, Ward T (2009). The transformation of violence in Iraq.. Brit J Criminol.

[25] Alwan HK, Jihad ST (2006). http://abcnews.go.com/International/print?id=2539854.

[26] United Nations Small Arms Review Conference (2006). http://www.un.org/events/smallarms2006/pdf/international_instrument.pdf.

[27] Coupland RM, Meddings DM (1999). Mortality associated with use of weapons in armed conflicts, wartime atrocities, and civilian mass shootings: literature review.. BMJ.

[28] Coupland RM, Samnegaard HO (1999). Effect of type and transfer of conventional weapons on civilian injuries: retrospective analysis of prospective data from Red Cross hospitals.. BMJ.

[29] Grossman D (1995). On killing: the psychological cost of learning to kill in war and society..

[30] Kahl CH (2007). In the crossfire or the crosshairs? Norms, civilian casualties, and U.S. conduct in Iraq.. Int Secur.

[31] Schmitt MN (2005). Precision attack and international humanitarian law.. International Review of the Red Cross.

[32] Walzer M (1977). Just and unjust wars..

[33] United Nations Security Council (2009). http://www.un.org/Docs/sc/sgrep09.htm.

[34] Pape RA (2005). Dying to win: the strategic logic of suicide terrorism..

[35] Gambetta D, Gambetta D (2006). Epilogue to the paperback edition.. Making sense of suicide missions.

[36] Pedahzur A (2005). Suicide terrorism..

[37] Moore S (29 November 2005) Killings linked to Shiite militias in Iraqi police force. Los Angeles Times. Available: http://www.latimes.com/news/nationworld/nation/ny-la-woiraq1129,0,1756028,full.story. Accessed 4 November 2010

[38] Roug L (2006). http://articles.latimes.com/2006/may/07/world/fg-civilians7.

[39] Carpenter RC (2006). Innocent women and children: gender, norms and the protections of civilians..

[40] United Nations Assistance Mission for Iraq (2010). http://www.uniraq.org/documents/UNAMI_Human_Rights_Report16_EN.pdf.

[41] Government of Iraq (2005). http://www.reliefweb.int/rw/RWB.NSF/db900SID/KHII-6CC44A?OpenDocument.

[42] Human Rights Watch (2003). http://www.hrw.org/en/reports/2003/10/20/hearts-and-minds.

[43] Freedberg SJ (2007). http://www.nationaljournal.com/njmagazine/nj_20071013_6.php.

[44] Matos RI, Holcomb JB, Callahan C, Spinella PC (2008). Increased mortality rates of young children with traumatic injuries at a US Army combat support hospital in Baghdad, Iraq, 2004.. Pediatrics.

[45] Coalition to Stop the Use of Child Soldiers (2008). Child Soldiers: Global Report 2008..

[46] United Nations OSRSG/CAAC (2008). http://www.un.org/children/conflict/english/index.html.

[47] Condra LN, Felter JH, Iyengar RK, Shapiro JN (2010). The effect of civilian casualties in Afghanistan and Iraq.. Working Paper 16152, NBER Working Paper Series.

[48] Daponte BO (2007). Wartime estimates of Iraqi civilian casualties.. International Review of the Red Cross.

[49] Harbom L, Sundberg R (2008). States in armed conflict 2007..

[50] Urlacher BR (2009). Wolfowitz conjecture: a research note on civil war and news coverage.. International Studies Perspectives.

[51] Iraq Family Health Survey Study Group (2008). Violence-related mortality in Iraq from 2002 to 2006.. N Engl J Med.

[52] Thoms ONT, Ron J (2007). Public health, conflict and human rights: toward a collaborative research agenda.. Confl Health.

[53] Johnson NF, Spagat M, Gourley S, Onnela J, Reinert G (2008). Bias in epidemiological studies of conflict mortality.. J Peace Res.

[54] Guerrero Serdán G (2009). http://www.rhul.ac.uk/economics/Research/WorkingPapers/pdf/dpe0901.pdf.

[55] Spiegel PB, Salama P (2000). War and mortality in Kosovo, 1998-99: an epidemiological testimony.. Lancet.

[56] Burnham G, Lafta R, Doocy S, Roberts L (2006). Mortality after the 2003 invasion of Iraq: a cross-sectional cluster sample survey.. Lancet.

[57] Kluger Y, Peleg K, Daniel-Aharonson L, Mayo A (2004). The special injury pattern in terrorist bombings.. J Am Coll Surg.

[58] Nasir K, Hyder AA, Shahbaz CM (2004). Injuries among Afghan refugees: review of evidence.. Prehosp Disast Med.

[59] Shapira SC, Adatto-Levi R, Avitzour M, Rivkind AI, Gertsenshtein I (2006). Mortality in terrorist attacks: a unique model of temporal death distribution.. World J Surg.

[60] Meddings DR (1997). Weapons injuries during and after periods of conflict: retrospective analysis.. BMJ.

[61] Bowley DM, Degiannis E, Westaby S, Mahoney PF, Ryan JM, Brooks AJ, Schwab CW (2005). Thoracic injury..

[62] Moseley TM (2003). http://www.globalsecurity.org/military/library/report/2003/uscentaf_oif_report_30apr2003.pdf.

[63] United States Central Command Air Forces (2008). http://www.afa.org/edop/2009/2004-08CFACCstats123108.pdf.

[64] United States Central Command Air Forces (2009). http://www.afcent.af.mil/shared/media/document/AFD-091103-001.pdf.

[65] Cordesman AH (2008). http://csis.org/files/media/csis/pubs/080318_afgh-iraqairbrief.pdf.

[66] WikiLeaks (2010). http://warlogs.wikileaks.org/.

[67] Cameron E, Spagat M, Hicks MH (2009). http://www.amsus.org/images/stories/podcast/2009BritishArmyReviewCBDAR.pdf.

[68] World Health Organization (2009). http://www.emro.who.int/iraq/surveys_imhs.htm.

